# Mother-child interaction: implications of chronic maternal anxiety and depression

**DOI:** 10.1186/s41155-019-0123-6

**Published:** 2019-04-11

**Authors:** Eloisa Pelizzon Dib, Flávia Helena Pereira Padovani, Gimol Benzaquen Perosa

**Affiliations:** Faculdade de Medicina de Botucatu, Universidade Estadual Paulista (UNESP), UNESP - Campus de Botucatu, Av. Prof. Mário Rubens Guimarães Montenegro, s/n, Bairro, Botucatu, SP 18618-687 Brazil

**Keywords:** Maternal mental health, Mother-child interaction, Chronic maternal depression, Chronic maternal anxiety

## Abstract

**Background:**

The literature has few studies on the quality of the mother-child interaction when mothers suffer from chronic anxiety and depression. This study aimed to compare characteristics of the interaction between 14-month-old children and their mothers who presented symptoms of chronic anxiety or depression with those of 14-month-old children and their mothers who did not present mental problems.

**Method:**

The sample consisted of 40 mother-infant dyads selected from a prospective cohort study. They were assessed using the State-Trait Anxiety Inventory and the Beck Depression Inventory, at three time points: during pregnancy and at 6 months and 14 months of the infant’s life. Three groups were formed: 10 mothers with symptoms of chronic anxiety, 8 mothers with symptoms of chronic depression, and a control group of 22 mothers without mental health problems. The mothers responded to a socioeconomic questionnaire, and then a 7-min episode of the dyad interaction was recorded and assessed using categories indicated in a dyadic interaction assessment protocol. This consisted of six categories that evaluate the behavior of the caregiver and four categories that evaluate the child’s behavior.

**Results:**

A significantly higher percentage of mothers with chronic depressive symptoms had not completed high school and did not live with a partner. When comparing the interaction behaviors of the three groups, mothers with symptoms of chronic depression were significantly less sensitive, were more disengaged, and showed less positive affect than those in the control group. They also engaged in significantly fewer stimulations and displayed more negative affect compared with both the control group and mothers with chronic anxiety symptoms. Anxious mothers presented greater intrusiveness compared with mothers in the control group. Regarding the children, those with mothers showing symptoms of chronic depression interacted significantly less than those with mothers showing symptoms of chronic anxiety and the control group.

**Conclusions:**

The results indicate that mother-infant interaction is most severely compromised among mother-infant dyads comprised of mothers with chronic depressive symptoms, compared with dyads of mothers with chronic anxiety symptoms and dyads of control group mothers without mental health problems.

## Background

From early infancy, favorable family context provides an important setting for the infant’s social–emotional development and well-being. This can alleviate the impact of adverse factors during periods of greater vulnerability for the child (Parfitt, Pike, & Ayers, 2014).

Among the various characteristics indicative of a healthy interaction, maternal responsiveness functions as a mediating factor and possible protection mechanism for development. The contingent response of the mother gives the child a sense of security that allows exploration and intervention in the environment, increasing the chances of good cognitive development and facilitating the acquisition of social skills (Alvarenga, Malhado, & Lins, 2014). However, the interactive situation is not only determined by maternal behavior, but it also relies on the child’s participation and is thus a bidirectional situation (Feldman, 2015; Parfitt et al., 2014).

The literature indicates several risk factors for good mother-child interaction: low socioeconomic status, low level of education, household density, fragile family bonds, conflicts, chronic illness of one of the family members, violence, and mistreatment (Cavalcante, Lamy Filho, França, & Lamy, 2017; Ribeiro, Perosa, & Padovani, 2014). In recent decades, maternal mental health problems, such as chronic depression and anxiety, have also been considered to be risk factors that can affect the initial relationship of the dyad and the child’s development, even when the maternal pathology is in remission (Chemello, Levandowisk, & Donelli, 2017; Goodman et al., 2011; Kaitz, Maytal, Devor, Bergman, & Mankuta, 2010).

High maternal anxiety has been associated with increased intrusiveness and decreased interactive behaviors among 3-month-old children, showing that this has an impact on the mother’s initial interactions with her child (Feldman et al., 2009). Studies of mother-infant interaction at 6 months and at the end of the first year of life have confirmed that mothers with high levels of anxiety present hyperarousal (Kaitz et al., 2010) and attachment impairment, together with less affection, less emotional warmth, and fewer positive feelings (Tietz, Zietlow, & Reck, 2014). Clavarino et al. (2010) observed that the mother’s anxiety had a close relationship with lower maternal sensitivity to the suffering of the child.

Studies on maternal depression are much more frequent. They show that puerperal depressive disorder, in addition to presenting characteristics typical of depressive symptoms, affects motherhood and the performance of the role of the mother. This leads to disinterest in the infant, negative feelings, and guilt due to being unable to provide adequate care, which affects the mother-infant interaction (Fernandes & Cotrin, 2013).

Depending on the severity and chronicity, anxious and depressive conditions can have more serious consequences for the development of and interaction with the child (Fernandes & Cotrin, 2013; Piccinini, Frizzo, Brys, & Lopes, 2014). Feldman et al. (2009) observed that both chronically anxious and depressed mothers impaired the interaction with the child. Clavarino et al. (2010) verified that children of chronically anxious mothers were 5.67 times more likely to have persistent attention problems than those whose mothers never experienced anxiety.

Regarding depression, studies have compared mothers with more persistent and recurrent depressive symptoms to those who experienced depression for short periods. They reported that the former presented less ability to respond in a sensitive and consistent manner, had less physical, visual, and verbal contact in interactions, and were less positive and affectively engaged than the latter. Those that were chronically depressed also set fewer limits and their disciplinary practices were more inadequate than non-depressed mothers (Petterson & Albers, 2001). The children of mothers with recurrent episodes of depression were less facially and vocally expressive, were more likely to develop insecure attachment, were less cooperative, and had greater difficulty controlling their anger and aggression (Gelaye, Rondon, Araya, & Williams, 2016).

According to Murray, Halligan, and Cooper (2010), chronic depression can have long-term effects because responsiveness and sensitivity toward the child are particularly impaired for long periods. A recent study by Granat, Gadassi, Gilboa-Schechtman, and Feldman (2017) compared the mother-child interaction in dyads without maternal mental health problems to dyads in which the mothers had chronic disorders: major depression or anxiety disorders. The chronically depressed mothers presented lower levels of gaze and touch synchrony. Moments of shared gaze were short followed by a quick snap in the mother’s gaze that led to gaze aversion in the child, social withdrawal, and less synchrony in mother-infant touch. The children of chronically anxious mothers presented worse social engagement than those of the control group; however, this was better than the children of depressed mothers. During play, an increase in the self-regulation behavior of the infants of chronically depressed mothers was also observed, possibly as a compensatory mechanism.

Although there is some research on the role of chronic conditions in the mother-child interaction, this knowledge remains limited, and longitudinal studies are particularly scarce. There are also few operational definitions for both chronic anxiety and depression, with no consensus among the available research. In a study by Petterson and Albers (2001), mothers who scored positively in at least two of the three evaluations performed —after birth and when the child was 28 and 50 months of age—were considered chronically depressed. Wojcicki et al. (2011) used two evaluations performed within 40 weeks of the birth to differentiate mothers with chronic depression, who scored positively in both evaluations, from those with episodic depression, who scored positively in only one. Regarding chronic anxiety, only two studies were identified in the literature. In both, these mothers scored positively in all three evaluations: at birth and at 6 and 9 months postpartum (Feldman et al., 2009; Granat et al., 2017).

In view of the above, there is a need for further studies regarding the effects of chronic anxiety and depression symptoms on the mother’s interaction with the child. These symptoms have been shown to affect short- and medium-term adaptation and development in the child. Thus, this study aimed to compare characteristics of the interaction between 14-month-old children and their mothers, who presented symptoms of chronic anxiety or depression, with those of 14-month-old children and their mothers, who did not present mental problems.

## Method

### Participants

A prospective cohort of pregnant women was attended by Brazilian National Health services in three cities in the State of São Paulo. We selected mothers who scored positively for depression or anxiety (trait and state) in all three evaluations: in the third-trimester gestational age and at 6 and 14 months postpartum.

Of the 139 mothers, 11 (8%) scored for depression and anxiety and 20 (14%) for trait and state anxiety, in all three evaluations. After attempts to contact them, some could not be found and others refused to participate; thus, 8 mothers with chronic symptoms of depression and anxiety and 10 who scored for trait and state anxiety in all three evaluations remained in the final sample. Following this identification, three groups were composed: the first (D) comprised mothers who scored for chronic depression and anxiety (*n* = 8), the second (A) comprised mothers who only scored positively for anxiety (trait and state) in the three evaluations (*n* = 10), and the third (C) comprised mothers who agreed to participate in the study and who did not score for anxiety or depression in any of the evaluations (*n* = 22).

### Instruments

In the initial cohort, self-evaluation instruments for anxiety and depression were used to evaluate maternal mental health.

The State-Trait Anxiety Inventory (STAI), developed by Spielberger, Gorsuch, and Lushene, translated and adapted for the Brazilian population by Biaggio and Natalício (1979), is a self-assessment instrument composed of 40 questions. These questions have four response alternatives each and are divided into two distinct subscales, state anxiety and trait anxiety. The STAI final score ranges from 20 to 80 points, with lower scores equating to lower levels of anxiety. For this study, the cutoff point used was that described by Caumo, Calvetti and Henriques (2016) and previously proposed by Faisal-Cury and Menezes (2006) in an epidemiological study on anxiety and depression in the pregnancy-puerperal cycle. Women who scored more than 40 points in each of the STAI subscales were classified as anxiety cases. Regarding the instrument’s psychometric qualities, its reliability in the test-retest was 0.74 for state anxiety and 0.83 for trait anxiety. The internal consistency alpha for female state anxiety was 0.88, and for female trait anxiety was 0.87 (Biaggio & Natalício, 1979).

The Beck Depression Inventory (BDI) is a self-assessment scale of behavioral manifestations of depression, which is non-diagnostic in purpose, translated and adapted to Brazil by Cunha (2001). It is composed of 21 categories of symptoms and attitudes, with four statements each, reflecting an increasing degree of depression severity (from 0 to 3). A total score is obtained by adding the individual scores of each category. A score above 18 was used as a cutoff point, as suggested by Oliver and Simmons (1984), which corresponds to symptoms of moderate and severe depression in the manual. Regarding the psychometric qualities, the internal consistency of the BDI was 0.84 and the test-retest correlation was 0.95 (*p* < 0.001) (Cunha, 2001).

The following instruments were used to collect data on the participants:

- Questionnaire on sociodemographic data covering the following: socioeconomic status—family income, occupation, parental education; environmental/social status—housing, marital status of the mother, number of children, child care support network, attendance at daycare; and the birth conditions of the child—gestational age and birth weight.
- A Dyadic Interaction Assessment Protocol, designed by the Childhood and Family Center (*Núcleo de Infância e Família*, NUDIF) of the Research Group on Childhood, Development and Psychopathology of the Federal University of Rio Grande do Sul (UFRGS) (Piccinini, Frizzo, & Marin, 2007), based on previous instruments. This protocol uses categories to evaluate the behaviors of the parents and 1-year-old children during their interaction. The instrument recommends that the interactions occur for a period of 7 min or more, that they are videotaped for later evaluation, and that the dyad is asked to play, as in everyday life. The equipment used included a video camera, age-appropriate toys, and the evaluation form. Maternal behavior was evaluated according to the categories defined in the instrument: sensitivity, cognitive stimulation, positive affect, negative affect, disengagement, and intrusiveness. The following categories were used to assess the child’s behavior: involvement, interaction, positive affect, and negative affect. Each category has five subcategories.

### Procedures

#### Data collection

After identification, telephone contact was made with the mothers eligible for the study, during which they were informed about the objectives and procedures and invited to participate. Meetings were scheduled at the residences of the women who agreed to participate, where, after all doubts were clarified, they signed a consent form and the sociodemographic data questionnaire was completed. Next, the mother was offered some toys appropriate for the child’s age and was asked to play and act naturally, as she would normally do with her child. The camcorder was switched on, and the researcher left the room for an average of 9 min. This was long enough to obtain the material needed for analysis, as recommended in the instrument.

#### Data analysis

To analyze the observational data, the initial minutes of the recording were discarded and interactive episodes of approximately 7 min were categorized by the researcher, minute by minute, in two steps (Piccinini et al., 2007). First, the researcher decided whether a given category was present in the interaction of the mother-child dyad. Next, per minute, each subcategory of the category was scored based on a 5-point Likert-type scale. For the final category score, per minute, the sum of the scores for all subcategories of the category was divided by the number of subcategories. The final score of each category, for the mother and child, was obtained from the sum of all the minutes.

To guarantee the quality of the operational definitions of the interactive behaviors, the concordance index between two evaluators was calculated. Twenty percent of the observations were drawn from the total (ensuring all three groups were represented), and two independent researchers, one of whom was the first author of this study, categorized the selected episodes. The Kendall index was calculated for each subcategory of the mother and child behavior. The vast majority of the categories had a concordance index higher than 0.70, while the category with the lowest index was maternal negative affect (0.68).

Descriptive analysis of the data was then performed, based on the calculation of frequency, percentage, or median, according to the nature of the data, followed by inferential statistical analysis. To investigate possible associations between the variables and differences between the groups, the chi-square test or Fisher’s exact test was used for categorical variables, and the Kruskal-Wallis test followed by the Dunn test was used for continuous variables. Spearman’s correlation test was used to evaluate possible correlations between the behaviors of the mother and child. The Statistical Package for the Social Sciences for Windows was used for the analyses. The results were discussed considering a 5% level of significance.

### Ethical aspects

This study was approved by a Research Ethics Committee under protocol no. 2014/606.856. The ethical guidelines proposed by Resolution 466/2012 of the National Health Council were rigorously followed.

## Results

The sample consisted of 40 children of approximately 14 months of age, the majority of whom were male (62.5%) and not attending daycare (65%). Most of the mothers were over 25 years of age (72.5%), lived with a partner (87.5%), had two or more children (65%), and had completed high school. Half of them worked outside the home and many had no help caring for the infant (65%). Regarding the fathers, most were over 25 years of age (85%), 37.5% had completed high school, and nearly all of them were employed (92.5%). The median household monthly income was R$1925.00, which corresponds to approximately twice the Brazilian minimum wage, which was R$ 954.00 in 2018.

Comparison of the sociodemographic characteristics of the groups verified they were similar, except in relation to maternal education level and the presence of a partner. A significantly higher percentage of mothers with chronic depressive symptoms had not completed high school and did not live with a partner (Table 1).

**Table 1:** Comparison of the sociodemographic and birth characteristics in the three groups

Characteristics	Control (*n* = 22)	Anxiety (*n* = 10)	Depression (*n* = 8)	*p* value
	*f* (%)	*f* (%)	*f* (%)	
Male sex	16 (72.7)	6 (60)	3 (37.5)	0.212
Preterm	1 (4.5)	1 (10)	1 (12.5)	0.579
Low weight	0 (0)	2 (20)	0 (0)	0.093
Daycare	9 (40.9)	3 (30)	2 (25)	0.740
Mother completed high school education	21 (95.5)	6 (60)	2 (25)	< 0.001*
Mother works	12 (54.5)	5 (50)	3 (37.5)	0.771
Mother has a partner	22 (100)	9 (90)	4 (50)	0.001*
Mother has help	4 (18.2)	4 (40)	1 (12.5)	0.350
Father completed high school education	15 (68.1)	7 (70)	3 (42)	0.491
Father works	21 (95.4)	10 (100)	6 (75)	0.144
Own home	13 (59)	5 (50)	6 (75)	0.564
	Med (Min–Max)	Med (Min–Max)	Med (Min–Max)	
Number of children	2 (1–3)	2 (1–4)	2 (1–3)	0.380
Mother over 25 years old	31 (17–39)	27 (19–35)	28 (18–37)	0.744
Father over 25 years old	31 (20–53)	29 (20–38)	34 (19–44)	0.175
Monthly income (R$)	2000 (788–4000)	1588 (788–3500)	844 (680–4000)	0.094

Chi-square test or Fisher’s exact test and Kruskal-Wallis test; * *p* < 0.05

*f* frequency, *%* percentage, *Med* median, *Min* minimum, and *Max* maximum values

Regarding the interactive behaviors, the median of negative affect, both maternal (med = 7.3, min = 7.0, max = 11.4) and infant (med = 7.4, min = 7.0, max = 13.0), was close to the minimum values, indicating that these characteristics were rarely present in the interactive episode.

Table 2 shows a high correlation between the behaviors of the mother and child. The more sensitive, stimulating, and positively affective the mothers were, the more the children were involved and integrated and presented more positive affect. Mother negative affect and child negative affect were rarely present. Maternal disengagement was associated with less interaction, more negative affect, and with less involvement and less positive affect in the child. The children of more intrusive mothers were less involved and interacted less.

**Table 2:** Correlation between mother and child behaviors in the interactive situation

Mother	Infant			
	Involvement	Interaction	Positive affect	Negative affect
	rho (*p* value)	rho (*p* value)	rho (*p* value)	rho (*p* value)
Sensitivity	0.68 (0.000)*	0.45 (0.004)*	0.41 (0.011)*	− 0.22 (0.193)
Cognitive stimulation	0.57 (0.000)*	0.44 (0.006)*	0.51 (0.001)*	− 0.17 (0.301)
Positive affect	0.67 (0.000)*	0.48 (0.003)*	0.52 (0.001)*	− 0.26 (0.109)
Negative affect	− 0.58 (0.000)*	0.27 (0.107)	− 0.16 (0.348)	0.43 (0.007)*
Disengagement	− 0.59 (0.000)*	0.41 (0.010)*	− 0.43 (0.006)*	0.36 (0.028)*
Intrusiveness	− 0.50 (0.001)*	0.32 (0.048)*	− 0.17 (0.299)	0.24 (0.145)

Spearman’s correlation; *p < 0.05

When comparing the interaction behaviors of the three groups, mothers with symptoms of chronic depression were significantly less sensitive, more disengaged, and showed less positive affect than those in the control group. They also showed significantly reduced stimulation and more negative affect compared with both the control group and the group with chronic anxiety symptoms. Control group mothers presented less intrusiveness than those of the groups with anxiety and depressive symptoms. The children of mothers with chronic depressive symptoms interacted significantly less than the children of those with anxiety symptoms and those of control group mothers (Table 3).

**Table 3:** Comparison of infant and maternal interactive behaviors in the three groups: control, chronic anxiety and depression

Interactive behaviors	Mental health			*p* value	C versus A versus D
	Control (*n* = 22)	Anxiety (*n* = 10)	Depression (*n* = 8)		
	Med (Min–Max)	Med (Min–Max)	Med (Min–Max)		
Maternal sensitivity	22.8 (14.7–28.9)	20.1 (12.0–25.6)	14.6 (9.1–19.7)	0.004*	C > D
Maternal cognitive stimulation	14.8 (9.6–29.6)	16.4 (8.7–22.0)	10.2 (7.3–17.4)	0.010*	C, A > D
Maternal positive affect	18.4 (9.6–22.6)	17.3 (11.0–19.8)	14.8 (10.0–16.0)	0.014*	C > D
Maternal negative affect	7.0 (7.0–9.6)	7.2 (7.0–9.8)	8.9 (7.0–11.4)	0.014*	C, A < D
Maternal disengagement	7.6 (7.0–20.8)	8.5 (7.0–15.8)	13.8 (9.5–27.8)	0.005*	C < D
Maternal intrusiveness	7.8 (7.0–20.0)	10.9 (7.0–13.2)	13.4 (7.8–18.0)	0.003*	C < A, D
Infant involvement	20,8 (15,8 – 26,5)	20.1 (10.9–7.0)	17.5 (12.5–22.0)	0.063	
Infant interaction	13.2 (9.0–23.0)	13.0 (8.6–22.0)	10.2 (7.4–14.2)	0.025*	C, A > D
Infant positive affect	9.3 (7.5–12.8)	9.3 (7.8–15.0)	8.5 (7.0–11.8)	0.392	
Infant negative affect	7.2 (7.0–13.0)	7.5 (7.0–10.0)	7.5 (7.0–8.8)	0.731	

Kruskal-Wallis test followed by the Dunn test for multiple comparisons; *p < 0.05

*C* control, *A* anxious, *D* depressive

## Discussion

The study participants lived in three cities in the State of São Paulo and presented a similar profile to that of other national studies evaluating pregnant and puerperal women (Beltrami, Moraes, & Souza, 2013; Morais, Lucci, & Otta, 2013).

Mothers with depressive symptoms presented lower levels of education and fewer of them lived with a partner compared with anxious mothers and the control group. The association between depressive symptoms, both prenatal and postnatal, and lower levels of education and socioeconomic status is recurrent in the literature, as are conflictive marital relationships and the lack of a partner (Gelaye et al., 2016; Morais et al., 2013).

In the interactive episode, reciprocal relationships were observed between the children and their mothers, confirming the mutuality and bidirectional character of the interaction during the first year of life (Cassiano & Linhares, 2015; Feldman, 2015). More sensitive, stimulating, and positively affective mothers had children that were more involved and integrated in the interaction. They displayed more positive affect with smiles, enthusiasm, and physical displays of affection and few episodes of negative affect, such as crying, negative vocalizations, anger, and hostility. Conversely, the children of more disengaged and intrusive mothers interacted less and were less involved. According to Piccinini et al. (2014), the intrusive mother does not respect the child’s autonomy, attempts to control their behavior with unnecessary interventions, and ends up impairing the experience of new acquisitions.

Corroborating other studies (Cassiano & Linhares, 2015; Ribeiro et al., 2014), behaviors of positive affect predominated in the recorded episodes, with few episodes of negative affect, on the part of both the mother and the child. It seems probable that minimal hostility and demonstrations of positive affect are characteristic of mother-child interactions in the age range studied (Cassiano & Linhares, 2015); however, since the observations occurred in the presence of a stranger, in particularly small rooms, this could have inhibited the emission of socially censored negative affect behaviors.

Concerning the relationship between mental health and the interaction, the initial hypothesis was confirmed. There were significant differences in the behaviors emitted during the episode of interaction by mothers with chronic depressive symptoms, those with chronic anxiety symptoms, and control group mothers. Mothers of the group with chronic depressive symptoms were significantly less sensitive, cognitively stimulated their children less, had more restrained demonstrations of positive affect, and presented more negative affect than those in the anxiety and control groups. When asked to play with their children as they would normally, they often stated that they did not play with them. This profile corresponds to the description of mothers with chronic depressive symptoms reported in the literature: mothers unresponsive or not attentive to the needs of the child and rarely ever involved in close exchanges (Herba, Glover, Ramchandani, & Rondon, 2016; Murray et al., 2010). However, the data also showed that mothers with chronic depressive symptoms were more intrusive than those in the other two groups. Servilha and Bussab (2015) observed that mothers with postpartum depression presented more intrusive behaviors and were less responsive, which made the contacts of these dyads asynchronous regarding affection and attention. The literature reports that mothers with symptoms of depression can present different styles of interaction, with some being disengaged, uninvolved in interactions, less communicative, and showing less synchronous interaction, although they are more empathic with the child (Cornish et al., 2005; Feldman et al., 2009). Others are intrusive, are less affective when dealing with their children, and are more hostile, impatient, and coercive (Murray et al., 2010; Piccinini et al., 2014). There is a third group of mothers who, despite the depression, still find pleasure in their relationship with the child, with a moderate amount of smiles, touches, and responsive behavior, thus impairing the interaction less (Fonseca, Silva, & Otta, 2010).

In contrast, mothers with chronic anxiety symptoms presented interactive behaviors similar to those of the control group. Studies related to the interactive characteristics of anxious mothers are still scarce and the results are inconsistent. Some authors reported that anxious mothers were less sensitive than those in the control group and more intrusive than depressed mothers (Feldman et al., 2009; Nardi, Rodrigues, Melchiori, Salgado, & Tavano, 2015). Kaitz et al. (2010) did not observe sensitivity deficits and intrusiveness in anxious mothers, only that they acted in a more exaggerated way with their 6-month-old children in the free play situation. Murray, Cooper, Creswell, Schofield, and Sack (2007) also observed small differences in some interactive situations of mothers with social phobia and their 10-week-old children, but not in others. In this study, although mothers with chronic anxiety symptoms scored higher in the intrusiveness category, the scores did not differ significantly from those of mothers without a mental disorder. However, the anxious mothers provided their children with more cognitive stimulation than the depressed mothers, with possible developmental gains, as previously observed by de Fraga, Linhares, Carvalho, and Martinez (2008).

The children of mothers with chronic depression symptoms interacted significantly less than those of the other two groups, in agreement with other studies (Feldman et al., 2009; Murray et al., 2010). Research on how children of depressed mothers behave during the interaction presents contradictory results. In her review article, Field (2010) concluded that disturbances in the child’s interaction with mothers who have symptoms of depression are a universal phenomenon. Other studies, however, have shown that children of depressed mothers can maintain the same quality of interaction as those of mothers without such symptoms (Fonseca et al., 2010; Ribeiro et al., 2014). In this study, the chronicity of the condition may have acted as an aggravating factor for the lower percentage of interactive behaviors of the children of depressed mothers (Stein et al., 2014). Similarly, Cornish et al. (2005) observed that mothers with chronic depression had a negative perception of their children, complained about having to take care of them, and that their hostile behaviors inhibited the child’s participation.

Although the children of mothers with symptoms of depression interacted less, there were no significant differences among the three groups regarding the other interactive characteristics of the children. Regardless of maternal mental health, the children of the three groups presented demonstrations of positive affect (positive vocalizations, smiles, laughter, hugs, and kisses), good involvement, and minimal negative affect, with rare demonstrations of crying, agitation, discontent, or hostility. The children’s involvement in the interaction, regardless of the mother’s mental health, with numerous demonstrations of positive affect, seems to indicate that the relationship between the mother with depression and the child has no linear causality. It is a complex process and the final outcome involves other variables, including the individual characteristics of the mother and of the child. It is also influenced by the surrounding environment, for example, the presence of the father, which was the case for the majority of the children in this study (Goodman et al., 2011; Mendonça, Bussab, Lucci, & Kärtner, 2015; Morais et al., 2013).

If evidence in the literature indicates that the lack of a partner aggravates the effects of the maternal depressive condition (Lovejoy, Graczyk, O’Hare, & Neuman, 2000; Murray et al., 2010; Stein et al., 2014), Goodman et al. (2011) showed that the presence of the father alleviated the overload related to child care of the depressed mother and could represent a potentially healthy alternative form of care for the child. Similarly, Parfitt et al. (2014) and Mendonça et al. (2015) observed that the presence of a father who was involved with the child could compensate for and reduce the impact of maternal depression on the child.

Another variable that may have favored the involvement of the children of the three groups in the interaction was their age at the time of the evaluation. Lovejoy et al. (2000) observed that although younger children were more sensitive to the effects of maternal disengagement, older children, around one and a half years of age, were less dependent on maternal initiatives and were already able to establish some social reciprocity. In pleasurable situations, as in free play, they initiated the interaction, compensating for the lack of maternal involvement and creating conditions for the mother to participate in the activities. In this study, faced with the disengagement of the mother, the children often used gestures and vocalizations to attract her attention. According to Feldman (2015), these behaviors can elicit more parental investment and better interaction and development conditions.

According to Morais et al. (2013) and Goodman et al. (2011), full comprehension of the involvement of children of mothers with depression in the interaction requires that we consider two other variables: resilience and temperament. The resilience of the child has shown compensatory effects in adverse environments when the child was cared for by mothers with chronic depression (Frizzo & Piccinini, 2007). Regarding temperament, there is strong evidence that parental care does not produce the same effects in all children; some are more susceptible to adverse effects and inadequate care from the parents, as they benefit more from protective environments (Parfitt et al., 2014). It has also been identified that children with easy temperaments are less vulnerable to inadequate parental care provided by mothers with depression (Goodman et al., 2011). Studies on the differential susceptibility of children to adverse factors and their role in the interaction, especially in relation to maternal depression, are relatively recent, and new research should assist in identifying children who are more vulnerable so that interventions can be more accurately directed.

Some limitations need to be taken into consideration in the analysis of the results. Regarding the size of the sample, despite various efforts, there were several losses in the selection of the participants and few mothers presented the stipulated criterion of chronicity. The small number of participants limited the possibility of some statistical analyses, such as structural equation modeling, which could provide a better understanding of the complementary effects between the variables, from a bidirectional perspective.

Regarding the interactive episode, the analyses were limited to a single episode for each dyad, which does not allow the behaviors observed to be classified as stable relationship styles. Some mothers with chronic depressive symptoms, on other occasions, could possibly show greater sensitivity toward the child, avoiding impairments in the interaction due to the depression. However, the instrument used to analyze the interaction showed a high level of concordance between observers and it was considered adequate for observing dyads with children of this age group, allowing the bidirectional relationship between the partners of the dyad to be captured.

Regarding maternal mental health, mothers with chronic depressive symptoms also scored high for anxiety. The overlap between symptoms of anxiety and depression is recurrent in the literature, which possibly reflects the psychometric limitations of the scales used for the evaluation, especially the STAI and BDI, which present high correlation (Andrade & Gorenstein, 2008). It can also be assumed that anxiety and depression are components of the same process of general psychological stress, since there is data showing that 30% to 58% of patients with symptoms of depression present comorbidity with symptoms of anxiety (Field, 2010). Since comorbidity in the group of mothers with depressive symptoms may have affected the comparison between the groups, a group of mothers with chronic anxiety symptoms without comorbidity was included. The results of this group reinforce the data that describe anxious mothers as more intrusive, though with a profile very close to that of mothers without mental health problems, which causes less impairment in the development of their children.

## Conclusions

In this study, we verified that mothers with chronic depressive symptoms experienced situations of greater vulnerability in relation to the other mothers. They presented lower levels of education and income, fewer worked, fewer had a partner, and all simultaneously presented chronic symptoms of anxiety. Corroborating data in the literature, mothers with chronic depression symptoms presented greater disengagement and intrusiveness, lower sensitivity, and fewer demonstrations of positive affect compared with mothers of the other two groups. Their children, in turn, interacted less. These data reinforce the view that maternal mental health is a public health problem requiring special attention. Actions should be directed toward the early identification of symptoms of maternal anxiety and depression, and interventions should aim to minimize the consequences, ensuring a better quality of life for the women and preserving the mother-child interaction.

Since maternal depression is a preventable and modifiable factor that can be acted on in the health sector, further research is recommended, especially longitudinal studies. These should aim to identify the possible impact of the interaction on the medium- and long-term socioemotional development of the child and would be of great relevance to develop interventions and improve mother-child health indicators.

## Abbreviations

BDI: Beck Depression Inventory
NUDIF: *Núcleo de Infância e Família*
STAI: State-Trait Anxiety Inventory
UFRGS: Federal University of Rio Grande do Sul
